# Supplementary material for: A Likelihood Approach for Real-Time Calibration of Stochastic Compartmental Epidemic Models
Source: PLoS Comput Biol. 2017 Jan 17;13(1):e1005257. doi: 10.1371/journal.pcbi.1005257 (PMC5240920; doi:10.1371/journal.pcbi.1005257)
Supplement: S1 File — (TAR.GZ) [file pcbi.1005257.s014.tar.gz › HSPH_Online-SI-Revision/MSS10/n1-severe/n1-severe_NEW2_table4.pdf]

1	newly 3 weeks spec	24.	5.76	{0., 14.}	76.5833	1.10424	newly 3 weeks cumul	81.	37.34	{8., 62.}	55.9753	0.462508	newly 5 weeks spec	28.	1.78	{0., 7.}	93.6429	1.98197	newly 5 weeks cumul	135.	42.39	{8., 83.}	68.6	0.63625	newly 50 weeks cumul	286.	44.22	{8., 91.}	84.5385	0.951366
2	newly 3 weeks spec	23.	9.97	{0., 21.}	63.2609	0.748485	newly 3 weeks cumul	90.	54.22	{14., 89.}	44.8667	0.342568	newly 5 weeks spec	19.	3.1	{0., 9.}	83.6842	1.37402	newly 5 weeks cumul	138.	63.53	{14., 111.}	54.6449	0.453814	newly 52 weeks cumul	204.	67.72	{14., 121.}	66.8039	0.598047
3	newly 3 weeks spec	38.	14.65	{1., 29.}	63.5	0.707376	newly 3 weeks cumul	102.	70.54	{30., 115.}	37.8235	0.255657	newly 5 weeks spec	22.	6.18	{0., 16.}	75.5455	1.13689	newly 5 weeks cumul	152.	86.19	{32., 141.}	46.1382	0.343132	newly 51 weeks cumul	261.	94.73	{33., 156.}	63.7433	0.533106
4	newly 3 weeks spec	24.	30.55	{20., 40.}	34.375	0.126745	newly 3 weeks cumul	94.	114.28	{86., 139.}	26.0213	0.0996185	newly 5 weeks spec	9.	18.28	{9., 25.}	110.222	0.31413	newly 5 weeks cumul	116.	155.84	{118., 183.}	36.8621	0.133726	newly 54 weeks cumul	163.	208.76	{156., 251.}	33.2147	0.124733
5	newly 3 weeks spec	23.	30.67	{20., 41.}	39.7826	0.142131	newly 3 weeks cumul	56.	109.91	{80., 139.}	96.3036	0.283606	newly 5 weeks spec	17.	20.63	{11., 29.}	39.8235	0.16713	newly 5 weeks cumul	100.	156.36	{106., 195.}	57.96	0.191849	newly 53 weeks cumul	148.	242.12	{129., 311.}	68.6757	0.221669
6	newly 3 weeks spec	43.	32.75	{23., 41.}	24.7674	0.132058	newly 3 weeks cumul	120.	99.36	{84., 113.}	17.45	0.0867045	newly 5 weeks spec	35.	28.	{19., 37.}	23.3143	0.123837	newly 5 weeks cumul	192.	157.6	{135., 181.}	18.2292	0.0909736	newly 50 weeks cumul	340.	370.	{323., 421.}	12.4882	0.0519898
7	newly 3 weeks spec	17.	21.62	{15., 30.}	35.0588	0.128048	newly 3 weeks cumul	65.	71.69	{58., 86.}	16.6615	0.0678342	newly 5 weeks spec	9.	17.05	{10., 26.}	94.1111	0.272501	newly 5 weeks cumul	90.	107.95	{85., 132.}	23.2333	0.0891251	newly 50 weeks cumul	125.	201.49	{142., 253.}	62.984	0.205417
8	newly 3 weeks spec	6.	12.66	{6., 20.}	115.	0.307951	newly 3 weeks cumul	53.	56.82	{38., 74.}	19.7736	0.0819351	newly 5 weeks spec	0.1	5.59	{1., 11.}	5506.	1.55417	newly 5 weeks cumul	55.	71.11	{46., 91.}	34.8182	0.12604	newly 54 weeks cumul	55.	81.39	{53., 109.}	51.4364	0.171935
9	newly 3 weeks spec	21.	8.88	{0., 26.}	71.619	1.1013	newly 3 weeks cumul	83.	45.18	{3., 97.}	55.9759	0.524436	newly 5 weeks spec	22.	3.11	{0., 10.}	88.7727	1.79094	newly 5 weeks cumul	119.	53.63	{3., 123.}	61.0504	0.62261	newly 50 weeks cumul	236.	60.27	{3., 154.}	75.4619	0.881656
10	newly 3 weeks spec	16.	22.9	{15., 30.}	45.625	0.152676	newly 3 weeks cumul	74.	85.1	{71., 98.}	16.9459	0.0664003	newly 5 weeks spec	16.	15.41	{9., 22.}	24.4375	0.115154	newly 5 weeks cumul	103.	120.11	{101., 140.}	18.2233	0.0712253	newly 50 weeks cumul	137.	176.68	{147., 208.}	29.3723	0.10855
11	newly 3 weeks spec	26.	21.8	{15., 31.}	23.7692	0.122548	newly 3 weeks cumul	70.	71.77	{58., 84.}	11.7857	0.0507291	newly 5 weeks spec	28.	17.52	{11., 24.}	37.5	0.224464	newly 5 weeks cumul	115.	109.49	{94., 127.}	10.4957	0.0480646	newly 47 weeks cumul	274.	229.11	{176., 280.}	18.0036	0.090417
12	newly 3 weeks spec	14.	3.34	{0., 8.}	78.7143	1.11761	newly 3 weeks cumul	54.	22.22	{8., 35.}	59.3333	0.457919	newly 5 weeks spec	7.	0.81	{0., 3.}	90.7143	1.56378	newly 5 weeks cumul	71.	24.85	{8., 43.}	65.3662	0.543225	newly 55 weeks cumul	74.	26.01	{8., 44.}	66.7162	0.558682
13	newly 3 weeks spec	28.	27.85	{4., 48.}	56.1071	0.337847	newly 3 weeks cumul	97.	99.07	{31., 150.}	48.7938	0.251009	newly 5 weeks spec	15.	20.37	{1., 38.}	99.9333	0.562401	newly 5 weeks cumul	134.	143.96	{35., 229.}	58.2687	0.295335	newly 50 weeks cumul	214.	228.28	{35., 389.}	69.1589	0.379053
14	newly 3 weeks spec	29.	9.84	{0., 20.}	67.931	0.774858	newly 3 weeks cumul	89.	53.95	{19., 93.}	43.2921	0.311775	newly 5 weeks spec	15.	3.93	{0., 11.}	75.9333	1.2125	newly 5 weeks cumul	126.	63.84	{19., 113.}	51.0794	0.396457	newly 55 weeks cumul	170.	70.38	{19., 135.}	59.3412	0.494577
15	newly 3 weeks spec	13.	8.53	{2., 16.}	45.1538	0.354137	newly 3 weeks cumul	54.	33.23	{17., 49.}	40.0556	0.260531	newly 5 weeks spec	3.	4.07	{0., 10.}	101.667	0.562371	newly 5 weeks cumul	64.	42.74	{22., 67.}	37.1563	0.242618	newly 49 weeks cumul	74.	51.66	{23., 79.}	38.9189	0.251624
16	newly 3 weeks spec	18.	17.58	{8., 28.}	33.4444	0.1699	newly 3 weeks cumul	97.	74.56	{47., 102.}	26.0619	0.144815	newly 5 weeks spec	13.	8.64	{3., 16.}	46.4615	0.305852	newly 5 weeks cumul	125.	96.18	{59., 131.}	26.112	0.143905	newly 55 weeks cumul	201.	113.34	{69., 164.}	44.01	0.272075
17	newly 3 weeks spec	13.	20.18	{11., 30.}	63.0769	0.198398	newly 3 weeks cumul	54.	80.55	{62., 102.}	50.2037	0.169943	newly 5 weeks spec	4.	10.96	{3., 21.}	181.5	0.397673	newly 5 weeks cumul	73.	105.76	{80., 142.}	46.6849	0.157524	newly 55 weeks cumul	87.	133.27	{86., 200.}	55.5517	0.173539
18	newly 3 weeks spec	19.	14.94	{0., 31.}	58.3158	0.562578	newly 3 weeks cumul	61.	58.58	{12., 106.}	46.2295	0.28178	newly 5 weeks spec	28.	7.33	{0., 20.}	74.5357	1.22345	newly 5 weeks cumul	119.	77.13	{13., 147.}	47.2353	0.378708	newly 55 weeks cumul	388.	94.29	{13., 201.}	75.6985	0.794595
19	newly 3 weeks spec	15.	15.17	{8., 22.}	30.7333	0.140716	newly 3 weeks cumul	71.	59.38	{45., 73.}	18.6197	0.0931814	newly 5 weeks spec	12.	9.55	{3., 15.}	37.5833	0.231044	newly 5 weeks cumul	93.	81.75	{62., 105.}	17.1935	0.0836797	newly 48 weeks cumul	186.	114.08	{81., 153.}	38.6667	0.225238
20	newly 3 weeks spec	7.	11.48	{6., 19.}	71.1429	0.214493	newly 3 weeks cumul	54.	45.2	{25., 67.}	30.2593	0.161105	newly 5 weeks spec	3.	7.02	{2., 12.}	146.	0.371464	newly 5 weeks cumul	64.	60.54	{37., 87.}	28.5313	0.134861	newly 48 weeks cumul	97.	80.51	{49., 114.}	24.4639	0.129134
21	newly 3 weeks spec	33.	16.3	{5., 27.}	51.8182	0.413927	newly 3 weeks cumul	107.	62.49	{36., 89.}	42.1589	0.270946	newly 5 weeks spec	12.	8.14	{1., 16.}	51.1667	0.472248	newly 5 weeks cumul	135.	82.19	{44., 124.}	40.1407	0.26253	newly 55 weeks cumul	223.	101.13	{44., 155.}	54.6502	0.398665
22	newly 3 weeks spec	12.	6.09	{0., 13.}	55.9167	0.696346	newly 3 weeks cumul	57.	30.52	{9., 50.}	47.4035	0.372523	newly 5 weeks spec	7.	2.18	{0., 7.}	75.1429	1.086	newly 5 weeks cumul	75.	35.95	{9., 60.}	52.3867	0.431263	newly 49 weeks cumul	81.	38.75	{9., 73.}	52.679	0.444384
23	newly 3 weeks spec	21.	9.95	{0., 21.}	61.1905	0.690597	newly 3 weeks cumul	88.	45.47	{17., 82.}	50.4432	0.374932	newly 5 weeks spec	16.	4.14	{0., 11.}	75.625	1.18319	newly 5 weeks cumul	122.	55.5	{18., 95.}	55.5082	0.439842	newly 51 weeks cumul	188.	64.07	{18., 117.}	65.9202	0.575965
24	newly 3 weeks spec	15.	5.65	{0., 15.}	67.9333	0.863813	newly 3 weeks cumul	68.	35.18	{9., 63.}	50.3529	0.394002	newly 5 weeks spec	6.	1.66	{0., 6.}	83.6667	1.25166	newly 5 weeks cumul	79.	40.03	{9., 74.}	51.6076	0.416004	newly 48 weeks cumul	103.	42.61	{9., 78.}	59.7573	0.510188
25	newly 3 weeks spec	9.	4.31	{0., 11.}	66.5556	0.799825	newly 3 weeks cumul	59.	28.65	{7., 49.}	53.5763	0.440066	newly 5 weeks spec	11.	0.99	{0., 5.}	91.	1.68215	newly 5 weeks cumul	83.	31.69	{7., 59.}	62.2048	0.547388	newly 46 weeks cumul	100.	32.85	{7., 66.}	67.15	0.617905
26	newly 3 weeks spec	16.	14.99	{1., 36.}	68.8125	0.492999	newly 3 weeks cumul	56.	65.7	{21., 130.}	58.5	0.255121	newly 5 weeks spec	15.	9.52	{0., 30.}	80.	0.965438	newly 5 weeks cumul	84.	87.54	{25., 191.}	57.4048	0.287339	newly 53 weeks cumul	169.	116.92	{25., 331.}	65.2781	0.446832
27	newly 3 weeks spec	8.	20.41	{1., 34.}	194.875	0.616215	newly 3 weeks cumul	28.	85.61	{18., 126.}	220.536	0.480164	newly 5 weeks spec	0.1	11.52	{0., 21.}	11474.	1.57299	newly 5 weeks cumul	28.	112.96	{20., 169.}	317.071	0.56944	newly 52 weeks cumul	28.	151.45	{20., 233.}	454.25	0.662723
28	newly 3 weeks spec	36.	38.34	{9., 55.}	37.4444	0.284514	newly 3 weeks cumul	124.	134.37	{86., 168.}	25.3468	0.123459	newly 5 weeks spec	31.	26.26	{0., 39.}	31.6129	0.405421	newly 5 weeks cumul	183.	193.08	{106., 243.}	27.9781	0.146859	newly 55 weeks cumul	256.	301.6	{106., 394.}	41.7031	0.205061
29	newly 3 weeks spec	15.	9.56	{2., 18.}	44.2667	0.325846	newly 3 weeks cumul	52.	38.29	{17., 58.}	32.9808	0.198421	newly 5 weeks spec	10.	4.69	{0., 10.}	57.9	0.640524	newly 5 weeks cumul	71.	50.27	{22., 79.}	35.3099	0.221565	newly 48 weeks cumul	150.	62.11	{25., 98.}	58.7	0.443657
30	newly 3 weeks spec	15.	15.75	{9., 22.}	26.3333	0.133609	newly 3 weeks cumul	70.	59.83	{50., 70.}	15.6714	0.0833189	newly 5 weeks spec	2.	10.94	{6., 15.}	449.	0.725508	newly 5 weeks cumul	78.	83.75	{72., 96.}	12.5513	0.0588807	newly 49 weeks cumul	112.	125.68	{99., 149.}	17.4107	0.0783237
31	newly 3 weeks spec	19.	19.88	{10., 30.}	34.5263	0.161003	newly 3 weeks cumul	55.	69.76	{47., 91.}	33.3455	0.122528	newly 5 weeks spec	24.	13.89	{4., 22.}	45.125	0.322252	newly 5 weeks cumul	98.	100.53	{69., 130.}	21.7041	0.0966067	newly 50 weeks cumul	218.	153.07	{95., 220.}	32.2982	0.190019
32	newly 3 weeks spec	37.	16.68	{0., 36.}	57.7297	0.707253	newly 3 weeks cumul	98.	56.1	{8., 102.}	45.7143	0.377178	newly 5 weeks spec	27.	11.19	{0., 30.}	66.7778	1.05765	newly 5 weeks cumul	151.	81.01	{8., 153.}	49.1589	0.441327	newly 52 weeks cumul	280.	119.64	{8., 290.}	61.35	0.613202
33	newly 3 weeks spec	31.	28.57	{14., 48.}	36.4194	0.180961	newly 3 weeks cumul	119.	108.32	{64., 174.}	33.1429	0.159066	newly 5 weeks spec	13.	17.01	{5., 32.}	66.5385	0.260938	newly 5 weeks cumul	150.	146.96	{81., 245.}	34.6267	0.156778	newly 55 weeks cumul	184.	199.21	{97., 334.}	42.0489	0.178516
34	newly 3 weeks spec	15.	7.32	{0., 17.}	60.9333	0.641257	newly 3 weeks cumul	64.	28.89	{7., 56.}	56.7969	0.470902	newly 5 weeks spec	10.	3.45	{0., 10.}	73.3	1.0733	newly 5 weeks cumul	87.	37.92	{8., 70.}	58.4598	0.512544	newly 47 weeks cumul	179.	44.52	{8., 98.}	75.1285	0.771087
35	newly 3 weeks spec	21.	12.85	{5., 21.}	44.619	0.305693	newly 3 weeks cumul	87.	56.41	{38., 76.}	36.6552	0.211063	newly 5 weeks spec	3.	5.14	{0., 13.}	114.	0.428019	newly 5 weeks cumul	98.	69.93	{42., 95.}	31.8469	0.180385	newly 55 weeks cumul	98.	81.2	{47., 120.}	28.4082	0.15184
36	newly 3 weeks spec	1.	2.36	{0., 7.}	238.	0.779271	newly 3 weeks cumul	11.	18.5	{5., 35.}	92.5455	0.287867	newly 5 weeks spec	0.1	0.31	{0., 1.}	384.	0.166513	newly 5 weeks cumul	11.	19.69	{5., 37.}	101.727	0.299086	newly 56 weeks cumul	11.	19.8	{5., 38.}	102.727	0.300226
37	newly 3 weeks spec	19.	4.69	{0., 11.}	75.4211	0.914379	newly 3 weeks cumul	60.	21.06	{6., 34.}	65.1	0.537573	newly 5 weeks spec	11.	2.18	{0., 7.}	82.7273	1.32179	newly 5 weeks cumul	82.	26.36	{7., 47.}	68.1463	0.596024	newly 46 weeks cumul	171.	30.77	{7., 58.}	82.0058	0.869388
38	newly 3 weeks spec	5.	11.59	{5., 20.}	137.8	0.335293	newly 3 weeks cumul	41.	53.	{37., 71.}	35.6585	0.128555	newly 5 weeks spec	3.	5.27	{1., 11.}	105.	0.34944	newly 5 weeks cumul	46.	65.83	{42., 86.}	48.0652	0.162671	newly 54 weeks cumul	51.	76.23	{45., 104.}	55.1961	0.180995
39	newly 3 weeks spec	20.	14.27	{5., 23.}	36.65	0.24031	newly 3 weeks cumul	64.	55.85	{33., 82.}	26.1094	0.136913	newly 5 weeks spec	14.	9.26	{0., 19.}	50.2857	0.492673	newly 5 weeks cumul	91.	76.39	{42., 111.}	27.3516	0.149753	newly 50 weeks cumul	151.	101.56	{43., 164.}	37.9073	0.24325
40	newly 3 weeks spec	21.	7.76	{0., 17.}	65.8095	0.77556	newly 3 weeks cumul	63.	44.2	{16., 75.}	38.6349	0.256305	newly 5 weeks spec	9.	2.66	{0., 9.}	81.1111	1.23619	newly 5 weeks cumul	89.	51.29	{17., 89.}	45.6517	0.333653	newly 56 weeks cumul	118.	55.42	{17., 103.}	54.7288	0.430343
41	newly 3 weeks spec	14.	12.8	{7., 19.}	27.7143	0.14757	newly 3 weeks cumul	52.	57.66	{43., 70.}	18.3462	0.0749394	newly 5 weeks spec	6.	5.89	{1., 11.}	51.1667	0.308559	newly 5 weeks cumul	63.	72.77	{53., 90.}	22.2063	0.08765	newly 53 weeks cumul	66.	88.33	{59., 115.}	38.8939	0.13797
42	newly 3 weeks spec	25.	25.7	{8., 39.}	41.2	0.224247	newly 3 weeks cumul	82.	88.78	{46., 116.}	29.1707	0.132483	newly 5 weeks spec	15.	19.7	{3., 31.}	68.	0.338217	newly 5 weeks cumul	118.	130.53	{60., 178.}	36.4831	0.166386	newly 53 weeks cumul	257.	240.19	{77., 366.}	42.8366	0.249536
43	newly 3 weeks spec	9.	10.43	{4., 18.}	48.1111	0.205592	newly 3 weeks cumul	28.	39.3	{23., 54.}	47.6429	0.164063	newly 5 weeks spec	9.	5.85	{0., 12.}	53.2222	0.486054	newly 5 weeks cumul	44.	52.82	{28., 79.}	36.5909	0.141839	newly 46 weeks cumul	120.	69.13	{30., 121.}	43.575	0.293367
44	newly 3 weeks spec	16.	11.84	{3., 22.}	42.125	0.293892	newly 3 weeks cumul	64.	54.86	{26., 83.}	27.875	0.152001	newly 5 weeks spec	5.	5.13	{0., 11.}	71.4	0.494249	newly 5 weeks cumul	85.	67.67	{30., 104.}	30.7176	0.176641	newly 54 weeks cumul	109.	76.82	{32., 116.}	34.6789	0.21699
45	newly 3 weeks spec	17.	12.37	{6., 19.}	34.0588	0.213808	newly 3 weeks cumul	55.	50.09	{35., 64.}	18.1273	0.0880451	newly 5 weeks spec	8.	6.66	{2., 11.}	37.25	0.251261	newly 5 weeks cumul	80.	65.47	{44., 87.}	22.1625	0.115661	newly 51 weeks cumul	157.	85.42	{57., 118.}	45.5924	0.281731
46	newly 3 weeks spec	36.	22.61	{13., 34.}	38.4167	0.234717	newly 3 weeks cumul	87.	76.87	{55., 98.}	19.4828	0.0962902	newly 5 weeks spec	13.	17.77	{6., 28.}	60.3846	0.242747	newly 5 weeks cumul	135.	115.57	{80., 151.}	21.1778	0.108606	newly 50 weeks cumul	318.	196.65	{117., 268.}	38.1918	0.231235
47	newly 3 weeks spec	0.1	2.83	{0., 6.}	2790.	1.04939	newly 3 weeks cumul	20.	23.5	{6., 42.}	50.2	0.226843	newly 5 weeks spec	0.1	0.6	{0., 2.}	658.	0.283659	newly 5 weeks cumul	20.	25.73	{6., 47.}	58.25	0.240785	newly 56 weeks cumul	20.	26.27	{6., 50.}	60.95	0.246815
48	newly 3 weeks spec	16.	10.32	{3., 18.}	44.125	0.332368	newly 3 weeks cumul	39.	39.7	{19., 59.}	28.8205	0.133683	newly 5 weeks spec	12.	4.94	{0., 12.}	64.8333	0.764567	newly 5 weeks cumul	57.	51.25	{25., 79.}	29.5965	0.155443	newly 50 weeks cumul	102.	64.57	{29., 110.}	40.6569	0.268168
49	newly 3 weeks spec	13.	6.29	{0., 12.}	56.3846	0.57346	newly 3 weeks cumul	64.	33.85	{13., 55.}	48.7031	0.348214	newly 5 weeks spec	9.	3.02	{0., 9.}	72.2222	1.078	newly 5 weeks cumul	90.	40.38	{17., 70.}	55.6667	0.424193	newly 54 weeks cumul	127.	44.43	{17., 80.}	65.0157	0.536835
50	newly 3 weeks spec	21.	9.58	{0., 21.}	64.381	0.775542	newly 3 weeks cumul	56.	43.12	{11., 78.}	43.0357	0.290012	newly 5 weeks spec	17.	4.16	{0., 15.}	78.9412	1.37895	newly 5 weeks cumul	91.	53.17	{11., 96.}	48.4286	0.381403	newly 52 weeks cumul	172.	58.06	{11., 121.}	66.7326	0.611028
